# Supplementary material for: Chromothripsis during telomere crisis is independent of NHEJ, and consistent with a replicative origin
Source: Genome Res. 2019 May;29(5):737–49. doi: 10.1101/gr.240705.118 (PMC6499312; doi:10.1101/gr.240705.118)
Supplement: Supplemental Material [file supp_gr.240705.118_Supplemental_file_1.zip › contigs/annotated_contigs/DB73/contig.2.DB73_length_265_mean_cov_25.6264150943.docx]

**DB73_length_265_mean_cov_25.6264150943**

```
TATCCCTGCAATGATCACCCAGGTGATGTACCACTTGTCAAGGCTCTGCCTACAGGGACATTGCGATGTATCTCTGCACTGATCACCTA
 >chrY:13460944-13461045 - E=1e-46 p=0e+00
GGTCATGTAACT|GAT|TGATGTAACTCTTGTCTAGGATCTGCCTAAAGGGACTTTGTGACATAACTCTGCACTGATCATCCAGGTGAT
                >chrUn_gl000216:145185-145324 - E=2e-63
GGGGCTTTTGTCTAGGCTCTGCCTAAGGGGGCATTGTGACATATCTCTGCACTGATCACCCAGGTGA|TGTAACTCTTGTATAAGCTCT

G
```
